# Multilayered Hemorrhage Secondary to Retinal Arterial Macroaneurysm Rupture: A Case Report and Review of Literature

**DOI:** 10.7759/cureus.23903

**Published:** 2022-04-07

**Authors:** Monia Mechrgui

**Affiliations:** 1 Ophthalmology, Primary Health Care Corporation, Doha, QAT

**Keywords:** sudden vision loss, retinal hemorrhage, anti-vegf, pars plana vitrectomy, optical coherence tomography angiography (oct-a), hypertension

## Abstract

Retinal arterial macroaneurysms represent an acquired vascular irregularity that is primarily observed in the elderly population. The high variability surrounding the clinical presentation of this condition makes the initial diagnosis challenging. Employing several diagnostic techniques including fundus fluorescence angiography, optical coherence tomography, and optical coherence tomography angiography ensures that any hemorrhages secondary to macroaneurysms rupture are identified promptly. This is crucial for appropriately managing the case and ensuring a good prognosis. Here, we present a case of a multilayered hemorrhage secondary to a ruptured retinal arterial macroaneurysm managed with a pars plana vitrectomy with gas tamponade and we aim to highlight the recent advancements in diagnosis and management of this condition.

## Introduction

Retinal arterial macroaneurysms (RAMs) represent an acquired vascular irregularity that is primarily associated with the geriatric population. These macroaneurysms are uncommon and have an estimated prevalence of one in 4500 people [1]. It has also shown a marked predominance in female patients and a strong association with arteriosclerotic vascular changes and hypertension [2]. These acquired saccular or fusiform dilatations are primarily present along the temporal branches in the retina, most prevalently at points of arteriovenous crossing or bifurcation [3].

The most reported symptom is sudden vision loss, due to hemorrhage or edema that impacts the macula; however, the clinical presentation of RAMs depicts a high degree of variation based on the current study [3]. Although the majority of macroaneurysm cases lapse without the need for intervention, in some instances, a multilayered hemorrhage may arise secondary to this condition. In such cases, medical management is deemed necessary and most commonly involves a surgical approach, such as a pars plana vitrectomy or pneumatic displacement of sub-macular hemorrhages [4]. Here, we present a case of multilayered hemorrhage secondary to RAM managed with a pars plana vitrectomy and gas tamponade.

## Case presentation

**Figure 1:**
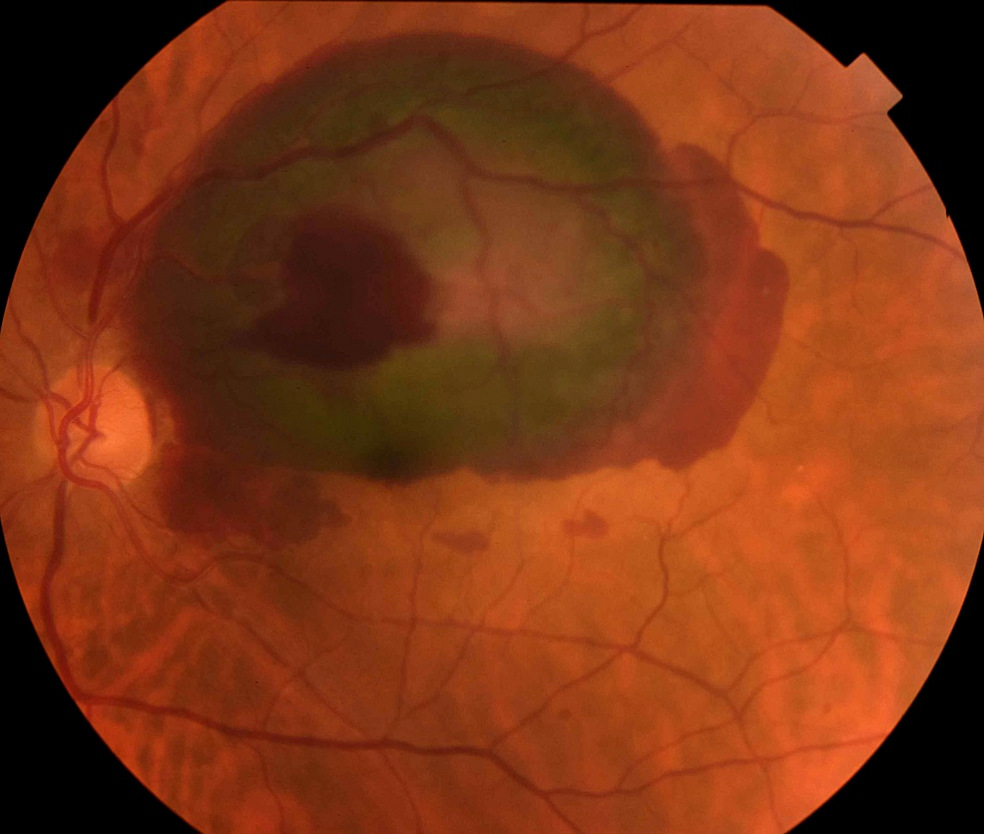
Left eye color fundus photography shows massive sub-retinal and pre-retinal hemorrhages in the supratemporal quadrant and the macular area.

A 67-year-old male presented to the emergency department following a sudden, painless loss of vision in his left eye over the previous 24 hours. The patient reported no significant medical history, no known hypertension, and no trauma that may have resulted in the onset of symptoms. His blood pressure measurement was 170/98 mmHg. On examination, his best-corrected visual acuity was 6/6 in his right eye and hand motion in his left eye. The anterior segments findings were normal. Goldmann applanation tonometry readings were 16 mmHg in both eyes. Dilated fundus examination of the right eye revealed a clear vitreous with a healthy optic nerve and mild attenuation of the vasculature. Assessment of the left eye showed a clear vitreous cavity, generalized attenuation of the arteries, and normal optic nerve head. Extensive intra-retinal and pre-retinal hemorrhages involving the supratemporal quadrant and the macula were noted. Fundus fluorescence angiography (FFA) and optical coherence tomography (OCT) were employed to confirm these findings. A multilayered hemorrhage in the left eye at presentation was confirmed (Figure 1).

**Figure 2:**
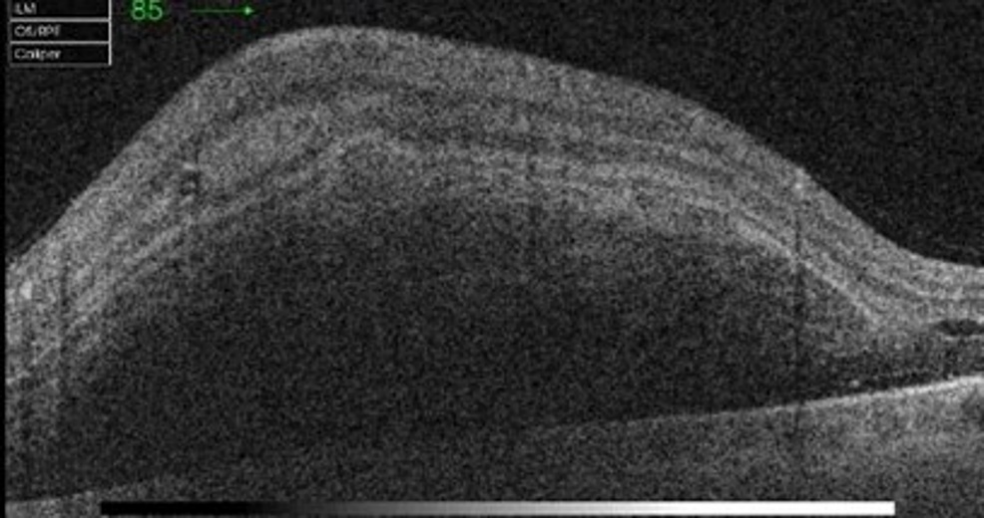
Left eye OCT scan shows massive sub-retinal and intra-retinal hyperreflectivity. OCT: optical coherence tomography

**Figure 3:**
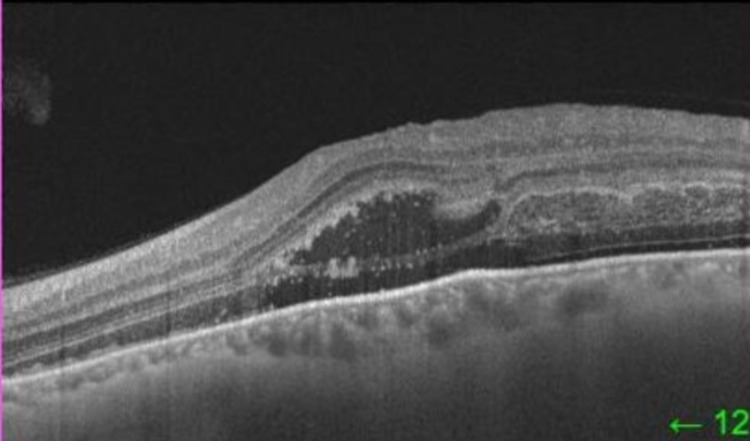
Left eye OCT scan shows sub-retinal hypo-reflectivity subsequent to the presence of serohematic fluid beneath the fovea. No choroidal abnormalities were noted. OCT: optical coherence tomography

**Figure 4:**
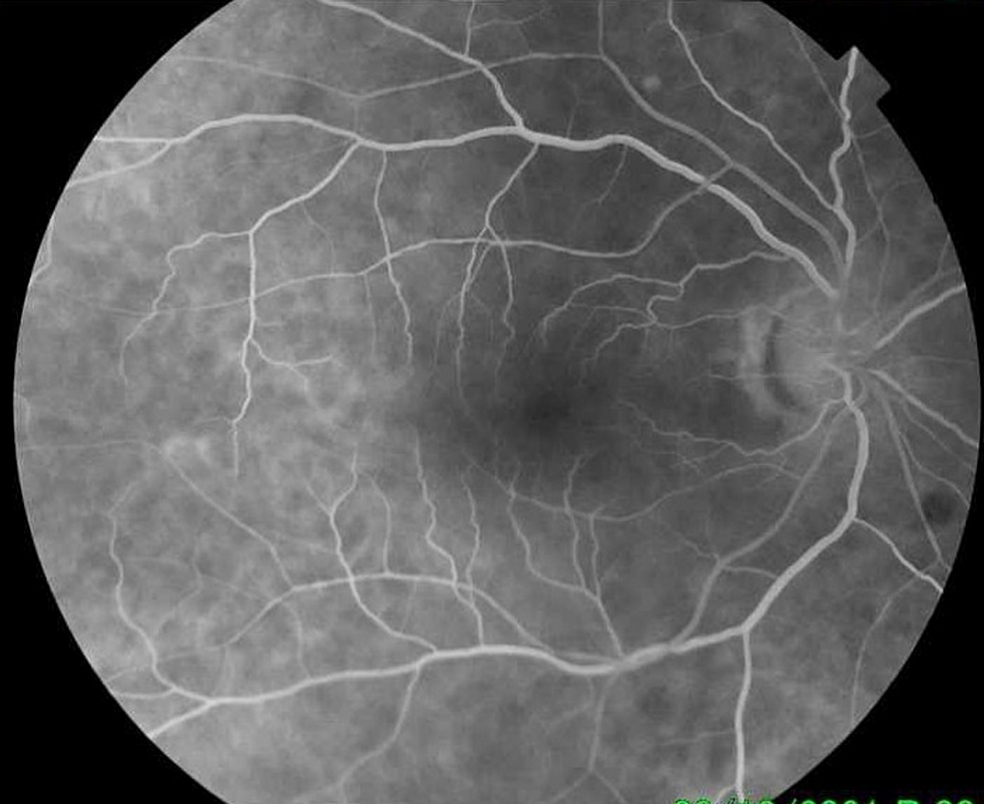
FFA of the right eye shows a normal angiogram. FFA: fundus fluorescence angiography

OCT of the left eye demonstrated dense sub-retinal and inner layer hyper-reflectivity with subsequent outer layer shadowing consistent with dense sub-retinal and intra-retinal hemorrhages (Figure 2). Sub-foveal hypo-reflectivity was noted as related to the presence of sub-retinal serohematic fluid (Figure 3). No macroaneurysms were visualized. FFA in the right eye was unremarkable (Figure 4).

**Figure 5:**
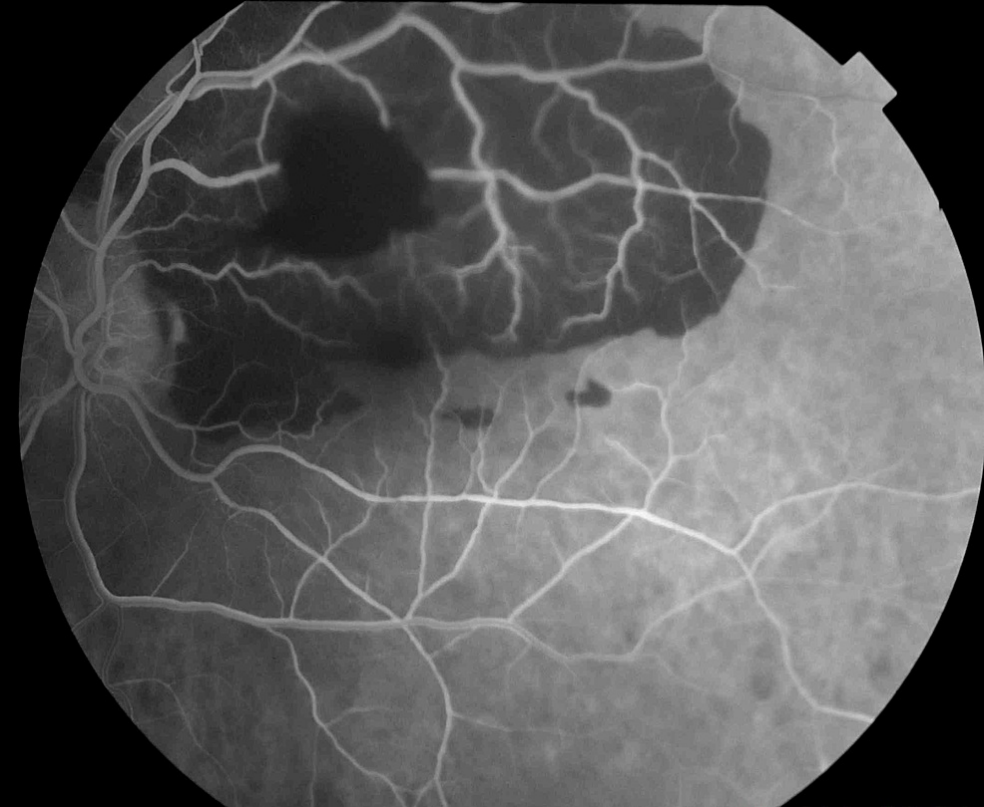
FFA of the left eye shows a total blockage of the fluorescein in the supratemporal quadrant and the macula. FFA: fundus fluorescence angiography

The left eye showed a complete blockage of fluorescein in the macular and supratemporal quadrant regions. The hemorrhage was predominantly sub-retinal; however, secondary pre-retinal hemorrhages areas were also observed. No leakage in the area of the presumed RAM was seen (Figure 5).

According to the clinical and paraclinical findings (normality of the right eye oct and angiogram and the absence of choroidal abnormalities in the left eye ), the patient was diagnosed with a multilayered hemorrhage secondary to RAM rupture. An immediate referral to the cardiology department was made, where essential hypertension was diagnosed, and treatment was initiated.

Due to the massive macular hemorrhage and in order to ensure a faster recovery with a better prognosis, surgical intervention in the form of a pars plana vitrectomy (PPV) with gas (C3F6) tamponade was decided. The surgery was performed 10 days after the onset of symptoms and was uneventful.

## Discussion

Clinical findings

The current study highlights the significant challenges faced during the initial diagnosis of RAM. The highly variable clinical presentation of this condition was discussed by Speilburg and Klemencic and described as a “masquerade” syndrome [4]. This was validated in a retrospective study of 40 patients which reported that 75% of the RAMs were misdiagnosed [5]. The most prevalent clinical presentation of RAMs in patients with macular involvement or a vitreous hemorrhage is an acute loss of vision. Macroaneurysms have various clinical presentations and they represent one of the few clinical entities that can produce sub-retinal, intra-retinal, pre-retinal, or vitreal hemorrhage or any combination of them.

Moreover, manifestations in a RAM rupture pose a challenge to diagnosis as they often mimic several other conditions, such as exudative age-related macular degeneration, Valsalva retinopathy, proliferative diabetic retinopathy, and complicated posterior vitreous detachment [4]. In some instances, this may present as a rounded dilation within the artery or as spontaneous pulsations [5].

Diagnosis

Diagnosis of retinal arterial macroaneurysm must be confirmed by complimenting clinical examination with imaging techniques such as FFA, OCT, and indocyanine green angiography (ICGA). FFA is highly beneficial in imaging concealed intra-retinal macroaneurysms and can even be used to differentiate between fusiform and saccular variations. While fusiform macroaneurysm shows early arterial filling, saccular dilatation has filling at later stages [4]. FFA additionally gives information regarding capillary microaneurysms, nonperfusion areas, intra-retinal microvascular abnormalities, and telangiectasias [6].

Although we conducted an FFA analysis on our case, we were not able to describe the nature of an aneurysm because of its late presentation in the form of a hemorrhage. However, it has been recommended to conduct indocyanine green angiography (ICGA) in cases where the nature of macroaneurysm cannot be confirmed because of the obscurity of hemorrhage [6]. RAMs show well-defined areas of hyper fluorescence with ICG angiography and ICG can pinpoint the exact location of the macroaneurysm in cases of dense hemorrhage, which may be useful in planning surgery or treatment [4].

OCT, in conjunction with dye-less angiography, provides an opportunity for diagnosis and management of retinal RAMs. A recent case report emphasized the importance of diagnostic procedures, including OCT and OCT angiography (OCTA), to prevent the misdiagnosis of RAM. Wilderson et al. described a 93-year-old male that presented with a similar clinical presentation to that of our patient; a sudden onset of vision loss over the previous 24 hours with no prior trauma, eye injury, or history of underlying health conditions that predispose to RAMs. Comprehensive diagnostic techniques enabled visualization of the morphological structure of the aneurysm, warranting a diagnosis of ruptured RAMs [7]. The use of these techniques is substantiated by Alnawaiseh et al. that discussed OCT A findings in patients with RAMs. In this study, three patients were retrospectively assessed, with all imaging data being comprehensively analyzed. The findings demonstrated that the diagnostic approach involving OCTA enabled the detection of macroaneurysms without the need for dye injection. Moreover, it was noted that the depth of the macroaneurysms in the patients’ retina, the exact localization of the macroaneurysms with regard to the main vessel, and the level of blood flow to the macroaneurysms could be determined through simultaneous observation of the OCT scans [8]. Recent work by Chang et al., as well as Lin and Hung, have particularly described the utility of OCT angiography in the management of retinal macroaneurysms [9,10]. They have reported that OCT angiography has a decisive role in the appropriate management of macroaneurysms as they allow clear visualization of the blood flow through it. This assists ophthalmologists to decide between photocoagulation, vascular endothelial growth factor inhibitors, or a combination of these two modalities for the treatment. Swept source-based OCT angiography for diagnosis, monitoring, and treatment of RAMs has a high degree of value as it provides a higher degree of magnification as compared to spectral-domain OCT [11,12]. 

Management

The current study proposes several management approaches for complicated RAMs, including moderate-intensity laser photocoagulation, sub-macular surgery, anti-vascular endothelial growth factor (VEGF) therapy, sub-retinal injection of recombinant tissue plasminogen activator (rt-PA), and PPV in patients with non-clearing, massive or breakthrough vitreous hemorrhage.

To evaluate the safety and efficacy of PPV to treat massive macular hemorrhage caused by ruptured RAM, Zhao et al. retrospectively reviewed the charts for eight eyes of eight patients treated by PPV. In each case, the pre-retinal/inter-lamellar hemorrhage was removed, and in three of the eight eyes, a sub-retinal hemorrhage was removed via a retinotomy after clot lysis using a tissue plasminogen activator [13]. Post-operative complications included a mild vitreous hemorrhage in two eyes, a macular hole in one, and a cataract in two. Our patient’s condition was treated with a PPV and gas (C3F6) tamponade due to the dense and extensive retinal hemorrhage with macular involvement and for faster visual recovery.

Intravitreal anti-VEGF injections have also been discussed for the management of complications. The effectiveness of this treatment was reported by Kishore. in a retrospective review of four patients over one year. The patients in this series were aged 68-91 years and were treated with intravitreal aflibercept injections of 2 mg for complications of retinal arterial macroaneurysm, including macula edema, sub-macular hemorrhage, and vitreous hemorrhage. A significant improvement in macular edema and vitreous hemorrhage incidence was observed, with excellent anatomical and visual results achieved in the majority of the cases. However, one patient presented with a thick sub-macular hemorrhage that was not successfully treated with this intervention [14]. Zweifel et al. investigated the effect of intravitreal anti-VEGF therapy for retinal macroaneurysms with macular exudation in ten patients, retrospectively. An improvement in corrected visual acuity was observed across all patients when compared to baseline, with central retinal thickness showing a marked decrease in cases with foveal involvement. Collectively, this suggests that intravitreal anti-VEGF therapy may offer a promising alternative treatment to moderate-intensity laser photocoagulation or surgical interventions in patients with RAMs [15].

Other methods, including sub-retinal injection of recombinant tissue plasminogen activator (rt-PA), have been discussed in the current study as a management approach in patients with a sub-macular hemorrhage resulting from a ruptured retinal arterial macroaneurysm. The surgical outcomes of small-gauge vitrectomy with rt-PA injection were discussed in a case series by Inoue et al. that assessed this intervention in 22 patients. The findings of this study demonstrated that the sub-macular hemorrhage was displaced from the foveal area in all patients after one week. Furthermore, a significant improvement in best-corrected visual acuity was observed from 1.41 logMAR units to 0.91 logMAR units after one month and 0.64 logMAR units at the final follow-up [16].

It is noteworthy to mention here that comprehensive management of RAMs should always deal with the ocular and systemic features of the disease [17]. In the majority of the cases, retinal macroaneurysms follow a benign course of thrombosis, fibrosis, and spontaneous resolution. In cases of non-complicated RAMs as well as those in which there is no threat to visual status, observation-based management protocol along with regular follow-up patterns as described by Speilburg and Klemencic should be adopted [4]. In asymptomatic cases, a regular follow-up every six months is essential till complete resolution. Additionally, macular sparring complications of retinal macroaneurysms with a low potential threat to vision should be followed closely for the first month. Afterward, a follow-up routine of one to three months is adequate till complete resolution.

## Conclusions

Retinal arterial macroaneurysm can result in hemorrhage to several retinal layers, including the pre-retinal, intra-retinal, and sub-retinal regions, making the initial diagnosis a significant challenge. Primary management of this condition relies on the management of the associated systemic risk factors; however, surgical intervention is often recommended, as was the case with our patient. Although not vastly reported, pars plana vitrectomy represents a valuable management option for patients with sight-threatening multilayered hemorrhage secondary to a retinal arterial macroaneurysm rupture.
